# Melanoma brain metastases treated with stereotactic radiosurgery and concurrent pembrolizumab display marked regression; efficacy and safety of combined treatment

**DOI:** 10.1186/s40425-017-0282-x

**Published:** 2017-10-17

**Authors:** Erik S. Anderson, Michael A. Postow, Jedd D. Wolchok, Robert J. Young, Åse Ballangrud, Timothy A. Chan, Yoshiya Yamada, Kathryn Beal

**Affiliations:** 1 Department of Radiation Oncology, Memorial Sloan Kettering Cancer Center, 1275 York Ave, New York, NY 10065 USA; 2 Department of Medicine, Memorial Sloan Kettering Cancer Center, New York, NY USA; 3 Department of Radiology, Memorial Sloan Kettering Cancer Center, New York, NY USA

## Abstract

**Background:**

Brain metastases are common in patients with metastatic melanoma. With increasing numbers of melanoma patients on anti-PD-1 therapy, we sought to evaluate the safety and initial response of brain metastases treated with concurrent pembrolizumab and radiation therapy.

**Methods:**

From an institutional database, we retrospectively identified patients with melanoma brain metastases treated with radiation therapy (RT) who received concurrent pembrolizumab. Concurrent treatment was defined as RT during pembrolizumab administration period and up to 4 months after most recent pembrolizumab treatment. Response was categorized by change in maximum diameter on first scheduled follow-up MRI. Lesion and patient specific outcomes including response, lesion control, brain control and overall survival were recorded and descriptively compared to contemporary treatments with RT and concurrent ipilimumab or RT without immunotherapy.

**Results:**

From January 2014 through December 2015, we identified 21 patients who received concurrent radiation therapy and pembrolizumab for brain metastases or resection cavities that had at least one scheduled follow-up MRI. Eleven underwent stereotactic radiosurgery (SRS), 7 received hypofractionated radiation and 3 had whole brain treatment (WBRT). All treatments were well tolerated with no observed Grade 4 or 5 toxicities; Grade 3 edema and confusion occurred in 1 patient treated with WBRT after prior SRS. For metastases treated with SRS, at first scheduled follow-up MRI (median 57 days post SRS), 70% (16/23) exhibited complete (CR, *n* = 8) or partial response (PR, n = 8). The intracranial response rates (CR/PR) for patients treated with SRS and concurrent ipilimumab and SRS without concurrent immunotherapy was 32% and 22%, respectively.

**Conclusions:**

Concurrent pembrolizumab with brain RT appears safe in patients with metastatic melanoma, and SRS in particular is effective in markedly reducing the size of brain metastases at the time of first follow-up MRI. These results compare favorably to SRS in combination with ipilimumab and SRS without concurrent immunotherapy.

## Background

Brain metastases are a common occurrence in patients with metastatic melanoma, occurring in up to 60% of patients [1, 2]. Although brain metastases portend a grave prognosis, recent advances in systemic therapy and treatment with radiation therapy have led to improved outcomes. Importantly, patients with good performance status and low number of brain metastases were found to have improved prognosis, in the era prior to immunotherapy [3]. Therefore, many patients with low number of melanoma brain metastases (MBM) undergo local therapy with either resection or radiation therapy (RT), typically in the form of stereotactic radiosurgery (SRS). Previous retrospective studies have shown SRS to be safe in combination with certain systemic therapies in melanoma, including ipilimumab, a monoclonal antibody to cytotoxic T-lymphocyte antigen-4 (CTLA-4) that enables T-cell activation and proliferation [4–7].

Pembrolizumab is a monoclonal antibody directed against programmed cell death 1 (PD-1), which is expressed on T-cells and is bound by its ligands PD-L1 and PD-L2 [8]. PD-L1 is expressed in both normal peripheral tissues as well as multiple human cancers. Its binding to PD-1 attenuates the function of the T-cells, and is one mechanism by which tumors evade immune surveillance [9, 10]. PD-1 blockade has been shown to have clinical efficacy in many tumors such as melanoma, non-small cell lung cancer, head and neck squamous cell carcinoma, urothelial bladder cancer, refractory Hodgkin’s lymphoma and mismatch repair deficient colorectal cancer [11–17]. Importantly, PD-1 inhibition has been shown to be both safe and efficacious when delivered with brain RT for metastases from multiple cancers including melanoma and non-small cell lung cancer [18–20].

Pembrolizumab treatment resulted in improved overall survival and less high-grade toxicity as compared to ipilimumab in a randomized controlled trial for metastatic melanoma, and has become increasingly used for treatment of metastatic melanoma [21]. Pembrolizumab alone has activity against melanoma brain metastases, but effects when combined with brain RT have not been reported [22]. As our institution has begun to increasingly incorporate pembrolizumab into metastatic melanoma treatment paradigms, we sought to examine early safety and efficacy outcomes of combined pembrolizumab with intracranial RT.

## Methods

### Patient identification and characteristics

From an institutional database, 21 melanoma patients were identified who received pembrolizumab concurrently with RT to brain metastases between January 2014 though December 2016. Pembrolizumab was delivered intravenously every 3 weeks, and RT occurred during or after pembrolizumab. For this study, concurrent pembrolizumab was defined as RT occurring at any point after first dose pembrolizumab up to 4 months after most recent pembrolizumab treatment. A 4 month concurrent period was chosen in an effort to capture all potential additive or synergistic effects of concurrent therapy, as persistent PD-1 occupancy (>70%) on T-cells by anti-PD-1 antibodies has been shown >2 months after a single treatment, despite serum half-life of only 12-20 days [12]. Identification of recent patients (2008 or later) who were treated with concurrent ipilimumab (RT up to 4 months after most recent ipilimumab) or who received RT without concurrent immunotherapy was performed from the same institutional database. Patient and treatment characteristics were recorded from the institutional database, and melanoma brain metastasis Graded Prognostic Assessment score was calculated as per prior reports [3].

### Brain radiation treatment

For SRS and hypofractionated radiation treatments, immobilization for treatment utilized the cranial Freedom SystemTM (CDR Systems, Alberta, Canada) with a custom head mold and an open face mask. Computed tomography (CT) images were acquired with 1.25 mm thickness on a Brilliance BigBore (Philips, Andover, MA) scanner at the time of simulation. Contrast enhanced SPGR (1 mm slice) and T1-weighted (3 mm slice) magnetic resonance (MR) images were fused to the CT images for target delineation. Brain metastases were treated with single fraction SRS, while post-operative and whole brain treatments were fractionated. Radiation dose was 20-21Gy for metastases less than 2 cm, 18Gy for metastases between 2 and 3 cm, and 30Gy in 5 fractions to metastases larger than 3 cm or a resection cavity. For each metastasis treated with SRS, a 2 mm expansion from enhancing lesion was used to create the planning target volume. SRS treatments included both static field 3-D conformal radiation therapy (3DCRT) plans and volumetric modulated arc therapy (VMAT) plans, with inhomogeneity in the range of 125-140% of prescription dose across all plans. Whole brain RT (WBRT) was 30Gy in 10 fractions (*n* = 2 patients) and 37.5Gy in 15 fractions (*n* = 1) if there was prior SRS, and was treated using 3 point mask and CT simulation.

### Toxicity and lesion response assessment

Toxicities were assessed with the Common Terminology Criteria for Adverse Events (CTCAE) 4.0 and were recorded via review of on-treatment notes and both medical oncology and radiation oncology follow-up notes. Each patient in the present study had a follow-up MRI for response assessment, typically between 6 and 8 weeks after completion of radiation therapy. Using these follow-up scans, lesion response was assessed via recorded maximum lesion diameter on planning and follow-up scans, while hemorrhage, recurrence and new brain metastases were noted. Complete response, partial response, stable response and progression designations were assigned to each lesion based on adapted criteria as proposed by the Response Assessment in Neuro-Oncology Brain Metastases working group (RANO-BM) [23]. Lesions that were not present on follow-up MRI were determined a complete response (CR), > 30% reduction in size a partial response (PR), > 20% increase in size progressive and the remainder stable.

## Results

### Patient characteristics and radiation treatments

From an institutional database of melanoma patients with brain metastases surveyed from January 2014 through December 2015, we identified 21 patients that received RT with concurrent pembrolizumab. All were treated after October 2014, and had at least 1 scheduled follow-up MRI available for review. Though inclusion criteria allowed RT up to 4 months after most recent pembrolizumab treatment, 17/21 (81.0%) patients had RT within 3 weeks of a pembrolizumab treatment. Patient characteristics are presented in Table 1. The patients had a median age of 67 at the diagnosis of brain metastases and median melanoma-specific Graded Prognostic Assessment (GPA) score of 3 at the time of analyzed RT. Thirteen of the 21 patients analyzed had undergone prior systemic therapy of any sort, with 10 having received prior CTLA-4 targeted therapy with ipilimumab, prior to change to pembrolizumab. Five patients had received BRAF-directed therapy (2 with concurrent MEK inhibitor), while the remainder had either BRAF wild-type tumors or had no available genetic information at the time of radiation therapy. Most patients had extracranial metastases (19/21), with the majority harboring lung or lymph node metastases, at the time of radiation treatment to the brain.

**Table 1:** Concurrent pembrolizumab and RT (*n* = 21)

Characteristic		Median (range) or *n* (%)
Patient characteristic		
Female		7 (33%)
Male		14 (67%)
Age at diagnosis of BM, y		67 (32-84)
Melanoma-specific GPA score		3 (1-4)
Karnofsky performance status		90 (80-90)
Median no. of BM treated w/SRS		1.5 (1-5)
Median BM diameter (maximum axial), cm		1.0 (0.2 - 2.4)
No. with non-brain metastases		19/21
No. with prior systemic therapy		13/21
	Ipilimumab	10/13
No. with BRAF directed therapy		5/21
No. with elevated LDH		3/12
Treatment characteristic		
No. with single fraction SRS		11 (52%)
	21 Gy, # lesions	19
	20 Gy, # lesions	1
	18 Gy, # lesions	3
No. with post-op RT (5 treatments)		7 (33%)
No. with whole brain RT		3 (14%)
Pembrolizumab dosage		
	2 mg/kg	18 (86%)
	10 mg/kg	3 (14%)
No. of pembro doses		6.5 (1-38)

Eleven patients underwent single fraction SRS to a median number of 1.5 lesions (23 total lesions treated) with a maximum number of 5 treated in 1 patient. Median lesion size was 1.0 cm (max 2.4 cm). The majority (19/23) were treated with 21Gy in a single fraction. Seven patients had hypofractionated radiation therapy, 6 of whom had resection of brain metastasis, to a dose of 30Gy in 5 daily fractions. Three patients received whole brain radiation therapy. One of the WBRT patients had received prior SRS treatment, approximately 20 months earlier.

### Toxicity and adverse events

Toxicities are shown in Table 2. There were no grade 4 or 5 toxicities. Fatigue was the most common adverse event; 14/21 patients reported at least grade 1 fatigue during or shortly after RT. The most severe toxicity was grade 3 edema surrounding treated metastases, which was observed in a patient who underwent WBRT with concurrent pembrolizumab. The increased edema caused significant left sided weakness and confusion requiring hospitalization but no operative intervention. Of note, this patient had undergone SRS 20 months prior to WBRT with pembrolizumab. Steroid treatment longer than 2 weeks was prescribed in 3/21 (14.3%) patients post-brain RT, and 10/21 (47.6%) had any steroid treatment. There was 1 asymptomatic intralesional hemorrhage detected on first follow-up MRI. Additional common toxicities included temporary mild confusion, or dermatologic and gastrointestinal symptoms while on concurrent treatment. When broken down into subsets of SRS, WBRT and hypofractionated treatments, the grade 1 toxicities were evenly distributed, while grade >/= 2 toxicity of CNS bleeding was seen after SRS treatment to multiple sub-centimeter metastases, seizure after SRS treatment to a 2.4 cm metastasis and neurologic dysfunction/perilesional edema/cognitive change in a patient with WBRT after prior SRS, as described above. Three additional cases of increased asymptomatic perilesional edema were seen after 2 SRS treatments and 1 WBRT treatment.

**Table 2:** Adverse events in patients receiving RT plus pembrolizumab (*n* = 21)

Adverse Event	Grade 1	Grade 2	Grade 3	Grade 4
Diarrhea/nausea	3			
Pruritis/Rash	2	1		
Cardiopulmonary				
Hepatitis				
Fatigue	14			
Headache	2			
CNS bleeding		1		
(from treated BM)				
Seizure	1	1		
Cognitive change	2	1		
Neurologic dysfunction	1	1		
Perilesional Edema		3	1	

### Metastases treated with SRS and concurrent pembrolizumab exhibit marked reduction in size on first follow-up MRI

**Fig. 1:**
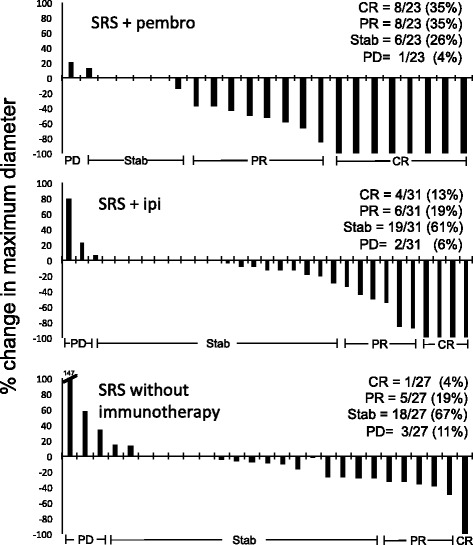
Complete and partial responses are frequently observed in metastases treated with concurrent SRS and pembrolizumab on first follow-up MRI. Waterfall plot showing percentage change in size of melanoma brain metastasis maximum diameter after SRS and the indicated systemic therapy at first follow-up MRI. (CR, complete response; PR, partial response; Stab, stable disease; PD, progressive disease; pembro, pembrolizumab; ipi, ipilimumab; SRS, stereotactic radiosurgery)

Eleven patients underwent SRS with concurrent pembrolizumab for metastatic melanoma, to a total of 23 lesions. All patients had at least 1 follow-up MRI available to assess lesion response. Follow-up scans for this group was performed at a median interval of 57 days post-SRS, with two scans occurring greater than 90 days from time of treatment. Of the 23 treated lesions, 8 exhibited a CR (35%), 8 exhibited a PR (35%) and 6 were stable (26%) on first planned follow-up scan (Fig. 1a; Table 3). One lesion increased in size from 1.5 cm to 1.8 cm (20% increase), which was the only progressive lesion. Of note, 2 patients had an extended time to scheduled follow-up scan (>90 days); 1 showed a stable response (1.6 cm to 1.8 cm), and 1 displayed a PR (0.7 cm to 0.1 cm). Lesions that demonstrated a CR, PR and stable response had median initial diameters of 0.8 cm, 1.0 cm and 1.1 cm, respectively.

**Table 3:** SRS Response rate at follow-up MRI

Treatment	Scan interval, d (range)	CR	PR	Stable	PD
SRS + pembro (*n* = 23)	57 (39-118)	8 (35%)	8 (35%)	6 (26%)	1 (4%)
SRS + ipi (*n* = 31)	53 (41-95)	4 (13%)	6 (19%)	19 (61%)	2 (6%)
SRS (*n* = 27)	51 (28-130)	1 (4%)	5 (19%)	18 (67%)	3 (11%)

Response assessment adapted from RANO proposal (Lin et al. 2015)

CR = complete response (disappearance of target lesion)

PR = partial response (lesion visible, ≥ 30% decrease in max diameter)

Stable = stable response (lesion visible, < 30% decrease OR <20% increase in max diameter)

PD = progressive disease (lesion visible, ≥ 20% increase in max diameter)

We were interested in comparing this response to that for patients who were treated with SRS and ipilimumab or without concurrent immunotherapy. From the same institutional database of patients with melanoma brain metastases, we identified 20 patients with a total of 31 lesions that were treated with SRS and concurrent ipilimumab, using the same inclusion criteria for concurrent treatment as with pembrolizumab (RT less than or equal to 4 months from most recent ipilimumab infusion). These lesions had an initial median diameter of 1.0 cm. On first follow-up scan, 13% (4/31) exhibited a CR, 19% (6/31) had a PR, 61% (19/31) were stable and 3/27 (11%) showed progression (Fig. 1b; Table 3). As a recent historical comparison, we also analyzed response of lesions in patients who did not have concurrent immunotherapy (15 patients with 27 treated lesions; median diameter 0.8 cm). In this group, 13 had no concurrent therapy, 1 had concurrent vemurafenib and one had recent temozolomide). In these patients, the majority of lesions were stable on first follow-up scan (67%; 18/27), while 5/27 showed PR (19%) and 1/27 showed CR (4%) as seen in Fig. 1c and Table 3.

### Treated brain metastases exhibit durable local control, though intracranial recurrence of new lesions is high

Median follow-up after date of brain RT and concurrent pembrolizumab was 276 days, and 11/21 treated patients had died due to progression of disease. The remaining 10 were alive with persistent evidence of systemic disease. In the 11 patients who had died, 13/14 brain metastases that were treated with SRS were controlled at the time of death (93%). At the time of death, 8/11 patients had suffered recurrence within the brain, and were treated with repeat SRS, surgery, or observation, as dictated by their clinical course. Six of the 8 patients who died after brain recurrence had causes of death that were most likely attributed to complications from progressive intracranial disease.

## Discussion

Brain metastases are a common site of disease in patients with metastatic melanoma, and effective treatment strategies are needed, given improving systemic outcomes with immunotherapy approaches. Previous work has shown that stereotactic radiosurgery (SRS) is an effective treatment modality that carries acceptable toxicity profile when delivered concurrently with ipilimumab [4, 5, 7]. Pembrolizumab is approved for treatment of advanced melanoma, and is associated with improved overall survival outcomes as compared to alternative contemporary therapies including ipilimumab [21, 24]. Previous reports have indicated that PD-1 inhibition with concurrent brain RT is safe and effective for patients with brain metastases from melanoma and non-small cell lung cancer [18–20]. We sought to assess toxicities and efficacy of pembrolizumab and concurrent RT to melanoma brain metastases.

We retrospectively identified a heterogeneous group of 21 patients from an institutional database, who received RT to the brain either concurrently with, or shortly after pembrolizumab treatment. In general, these were patients who had displayed progression on multiple prior courses of systemic therapy. In the latter period of investigation, an increasing number had been treated with pembrolizumab as a first-line agent for metastatic melanoma, given recent randomized trial data [21]. Treatment for brain metastases was SRS for the majority of patients, while a subset had resection of symptomatic metastases and received radiation to the post-operative cavity. This treatment paradigm followed institutional practices, and was largely dependent upon resectability of symptomatic tumors, symptom severity, and performance status of the patients.

**Fig. 2:**
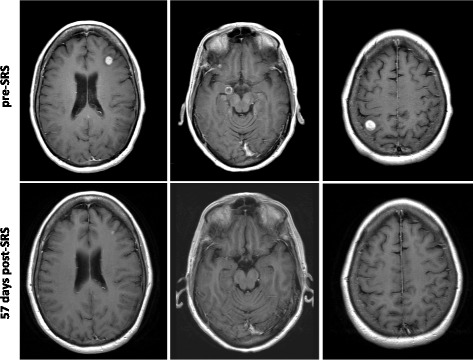
Melanoma brain metastasis response to concurrent SRS and pembrolizumab. Shown are representative responses in a single patient who received SRS with concurrent pembro to multiple brain metastases. T1, gadolinium-enhanced axial images of three treated lesions on pre-SRS planning MRI and first follow-up MRI illustrate PR in left panels and CR in middle and right panels with no contrast-enhancing focus visible on follow-up MRI

The striking finding from the current study is that metastases treated with SRS and concurrent pembrolizumab displayed a marked regression after treatment (representative response in Fig. 2). We show that >30% of lesions displayed a complete response at first planned follow-up scan, at a median of approximately 2 months after radiation treatment. Although our group is small, this response suggests an additive or potentially synergistic benefit to concurrent SRS and pembrolizumab for RT treated lesions. Previous reports have shown that systemic treatment with pembrolizumab alone has resulted in partial response in a modest fraction of brain metastases for melanoma patients (4/22; 22%) [17], and SRS alone has resulted in stable or reduced size of melanoma brain metastases in no more than 60% of treated lesions [25]. To our knowledge, the degree of complete response shown here on first follow-up MRI for melanoma metastases treated with SRS has not been previously reported; Yaeh et al. reported a 30.6% CR rate in radioresistant brain metastases (including melanoma, sarcoma and RCC) at a median follow-up of 6 months [26] by RECIST criteria.

Our observed effect may have a correlation to the initial size of the lesion, as those with complete response had median initial size of 0.8 cm, while partial responders and stable responders had median initial sizes of 1.0 cm and 1.1 cm, respectively. In our descriptive comparison to patients treated with SRS and concurrent ipilimumab or with SRS without immunotherapy, the responses to SRS and pembrolizumab appeared favorable. Past studies have suggested that concurrent checkpoint blockade with CTLA-4 inhibition and SRS to brain metastases has durable local control of treated lesions [4, 5, 7], though not all reports have shown an enhanced benefit to SRS with concurrent ipilimumab [6]. In this study, we found that 93% of treated lesions were controlled at the time of death after SRS and concurrent pembrolizumab, similar to the prior reports of durable control.

Overall, pembrolizumab and brain radiation therapy appeared to have acceptable acute toxicity. Specific acute toxicities that were observed were most commonly fatigue and headache, similar to what has been reported for brain radiation treatment alone [27]. Steroid use was low in these patients, with 3 patients requiring more than 2 week steroid taper, and <50% receiving any steroid treatment at all. Notably, the overall rates of systemic toxicities including diarrhea, nausea, rash or hepatitis were lower that what was previously reported for ipilimumab and SRS [4]. This result is consistent with pembrolizumab’s generally more well tolerated side effect profile as compared with CTLA-4 antibodies [13, 21].

Grade 3 toxicity was seen in 1 patient, which was recorded as grade 3 peri-lesional edema resulting in confusion, weakness and hospital admission. He had received whole brain radiation therapy with concurrent pembrolizumab, and had received SRS to multiple lesions in a previous treatment course approximately 20 months earlier. While this patient did not require operative management, he ultimately died due to progression of disease, approximately 90 days after radiation treatment. This outcome is notable, as there has been case report of increased edema surrounding melanoma brain metastases with pembrolizumab alone [28]. Of the 3 additional patients who experienced increase in peri-lesional edema after brain RT on follow-up MRI, two had undergone SRS to metastases and one had WBRT; all were asymptomatic. These data suggest that larger RT targets with concurrent pembrolizumab may be associated with increased risk of edema, which must be prospectively studied. Finally, there was no observed radiation necrosis on the initial follow-up MRI scans examined here. Since radiation necrosis can be a significant late toxicity to SRS treatment, further data must be examined to determine the long-term rates of radiation necrosis after concurrent pembrolizumab and SRS.

**Fig. 3:**
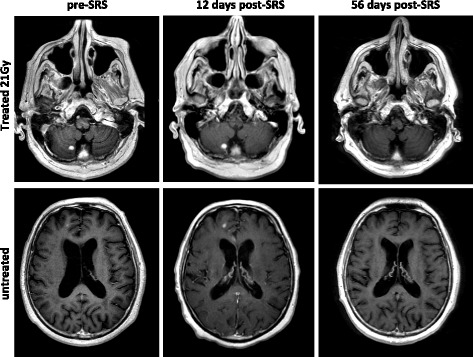
Untreated lesion response after SRS and pembrolizumab. In a patient who underwent SRS to a right cerebellar melanoma brain metastasis, a CR in an untreated right frontal lesion was observed. The right frontal lesion was not initially treated, as it was not clearly pathologic on pre-SRS scan. In an unplanned MRI 12 days post-SRS, the untreated lesion is more convincing of a metastasis. On planned follow-up MRI, both treated and untreated lesion display a CR

One intriguing hypothesis surrounding treatment of melanoma metastases with SRS and concurrent checkpoint blockade is the induction of response in untreated lesions [29]. While the overall numbers of patients in this study were low, we did observe 1 out of field response to concurrent pembrolizumab and SRS (Fig. 3). In this case, a patient who received pembrolizumab as first-line treatment for metastatic melanoma underwent SRS to a solitary right cerebellar metastasis. One punctate area of enhancement in the right frontal lobe was not treated as imaging evidence was not conclusive on planning MRI. He underwent an unplanned MRI of the brain 12 days post-SRS to rule out acute stroke, which showed interval growth of the untreated lesion, with characteristics more convincing of metastasis. Importantly, the treated lesion showed no significant treatment-associated changes at this time interval, including no increased hemorrhage or edema. On planned follow-up MRI at 56 days follow-up, both the treated and untreated lesion exhibited complete response. While such a finding could be attributed to pembrolizumab alone, further study is necessary to examine the contribution of abscopal response after concurrent SRS. Despite this intriguing response, it remains nonetheless concerning that despite effective treatment of the irradiated lesions, many patients still had intracranial progression with new lesions arising after combined RT and pembrolizumab.

While survival length is not well assessed in a retrospective review of a heterogeneous group of patients, most of whom were selected for SRS, the overall follow-up is encouraging in a heavily pre-treated group of patients. Others have suggested that previously described prognostic systems, including the graded prognostic assessment (GPA) for patients with brain metastases, are less applicable in the era of checkpoint blockade [30, 31]. Indeed, nearly 50% survival at median follow-up of over 9 months in a group with median melanoma-specific GPA of 3 exceeds that which would have been predicted from previous prognostic data.

The limitations of a small retrospective study such as this are numerous, including a mixed patient population, notably with respect to prior and current systemic treatment. Additionally, toxicity and follow-up data is retrospectively gathered from chart review, as opposed to prospective collection, which limits comprehensive toxicity analysis. Furthermore, the assessment of treatment response in the era of immunotherapy is not yet well-established, especially on longer-term follow-up scans. Regardless, the findings here are informative that brain metastases display impressive response and that the safety profile is similar to that which has been previously described for brain RT and CTLA-4 inhibition. Prospective studies to examine the long-term efficacy of combined treatment with pembrolizumab and brain RT are needed, and will more comprehensively examine the provocative findings described here.

## Conclusions

Concurrent pembrolizumab with SRS appears safe and effective in markedly reducing the size of melanoma brain metastases at the time of first follow-up MRI. These results compare favorably to SRS in combination with ipilimumab and SRS without concurrent immunotherapy. Further work is necessary to evaluate the safety and efficacy of larger volume radiation therapy, including WBRT, with concurrent pembrolizumab. Future studies will evaluate long term outcomes with respect to local control and late toxicity of combined treatment.

## Abbreviations

CNS: Central nervous system
CR: Complete response
CTLA-4: Cytotoxic T-lymphocyte antigen-4
Gy: Gray
MBM: Melanoma brain metastases
MRI: Magnetic resonance imaging
PD-1: Programmed cell death 1
PR: Partial response
RT: Radiation therapy
SRS: Stereotactic radiosurgery
WBRT: Whole brain radiation therapy
